# Association between Serum Soluble Urokinase-Type Plasminogen Activator Receptor Level and Arterial Stiffness in Chronic Hemodialysis Patients

**DOI:** 10.3390/jpm13030470

**Published:** 2023-03-04

**Authors:** Wei-Chen Lin, Tsung-Jui Wu, Chih-Hsien Wang, Yi-Jen Hsieh, Bang-Gee Hsu

**Affiliations:** 1 Division of Nephrology, Department of Internal Medicine, Hualien Tzu Chi Hospital, Buddhist Tzu Chi Medical Foundation, Hualien 97002, Taiwan; 2 Division of Nephrology, Department of Medicine, Hualien Armed Forces General Hospital, Hualien 971051, Taiwan; 3 Division of Nephrology, Department of Internal Medicine, Tri-Service General Hospital, National Defense Medical Center, Taipei 114202, Taiwan; 4 Institute of Medical Sciences, Tzu Chi University, Hualien 97004, Taiwan; 5 School of Medicine, College of Medicine, Tzu Chi University, Hualien 97004, Taiwan

**Keywords:** carotid–femoral pulse-wave velocity, soluble urokinase-type plasminogen activator receptor, hemodialysis, arterial stiffness

## Abstract

Cardiovascular diseases (CVDs) remain a significant cause of death in hemodialysis (HD) patients. To explore their associations, we examine the role of soluble urokinase-type plasminogen activator receptor (suPAR) in arterial stiffness in chronic HD patients. From June to August 2020, we recruited 135 chronic HD patients. The arterial stiffness group included patients with a carotid–femoral pulse-wave velocity (cfPWV) of >10 m/s. Fifty-five HD patients (40.7%) were in the arterial stiffness group. They had a higher prevalence of diabetes (*p* = 0.001) and hypertension (*p* = 0.039), were older (*p* = 0.007) and had higher aortic systolic blood pressure (*p* = 0.034), brachial systolic blood pressure (*p* = 0.025), glucose (*p* = 0.019), C-reactive protein (*p* = 0.039), and AIx75 (*p* = 0.003) and suPAR (*p* < 0.001) levels than the control group. After we performed multivariable logistic regression analysis, except age and glucose, serum suPAR (odds ratio [OR]: 2.05; 95% confidence interval [CI]: 1.48–2.70, *p* < 0.001) was independently associated with arterial stiffness in chronic HD patients. In the multivariable linear regression analysis, suPAR positively correlated with cfPWV (β = 0.475, *p* < 0.001) and could serve as a biomarker for arterial stiffness development in patients undergoing HD.

## 1. Introduction

Cardiovascular (CV) diseases remain the leading cause of death worldwide [1]. Several traditional risk factors have been identified for CV diseases, including age, smoking, diabetes mellitus (DM), hypertension, dyslipidemia, and chronic kidney diseases (CKDs) [2]. Other nontraditional CV risk factors, including left ventricular hypertrophy, serum albumin, hemoglobin, phosphates, and urates, are important in patients with CKD [2]. According to previous studies, CVD risk and mortality start to increase at stage 3 CKD [3,4] and become even more significant at stage 5 CKD and end-stage renal disease (ESRD) [5]. These patients with CKD were more vulnerable to nontraditional risk factors of CV diseases, and these nontraditional factors induce arterial stiffness, arterial calcification, and atherosclerosis [6]. Numerous studies have indicated that arterial stiffness, measured by carotid–femoral pulse-wave velocity (cfPWV), is related to worse CV outcomes and higher risk [7]. Recently, a meta-analysis revealed that participants with high cfPWV by 1 standard deviation (SD), 1 m/s, and cutoff points have a high pooled relative risk for CV events and CV mortality [8].

Although both invasive and non-invasive imaging studies are essential to CV diseases diagnosis, several serum markers are under investigation. During inflammation, soluble urokinase plasminogen activator receptor (suPAR) is detached from the endothelia, fibroblasts, and immune cells [9]. It promotes plasmin activation and chemotaxis, leading to atherosclerosis [10]. Some reports have revealed its role in estimating the severity and prognosis of heart failure and coronary artery diseases [11,12]. However, this relationship is often affected by non-atherosclerotic inflammation. Patients with ESRD often suffer from both chronic inflammation and atherosclerosis [13]. To evaluate the role of suPAR in the development of arterial stiffness among patients with ESRD, we aimed to examine the relationship between cfPWV values and suPAR in patients on maintenance hemodialysis (HD).

## 2. Materials and Methods

### 2.1. Patients

**Figure 1:**
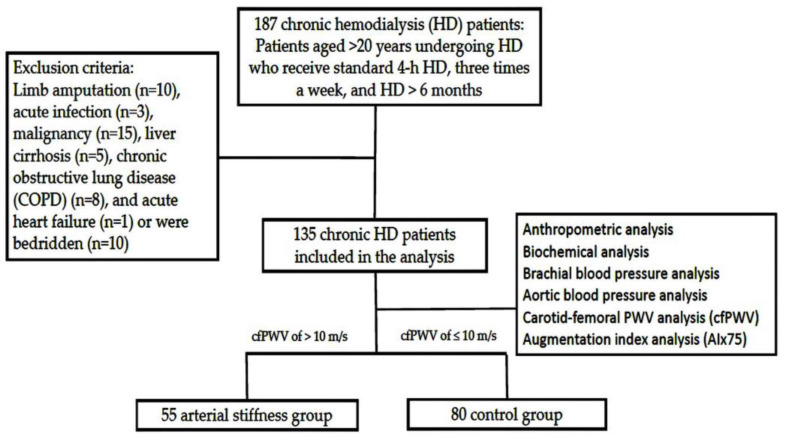
Flow chart of this study.

This study recruited patients undergoing HD between 1 June 2020 and 31 August 2020 in the HD unit of a medical center in Hualien, Taiwan. Patients aged >20 years undergoing HD who receive standard 4 h HD, three times a week, and HD > 6 months, were screened for enrolment. Patients who had a limb amputated (*n* = 10), acute infection (*n* = 3), malignancy (*n* = 15), liver cirrhosis (*n* = 5), chronic obstructive lung disease (COPD) (*n* = 8), and acute heart failure (*n* = 1) or were bedridden (*n* = 10) were excluded. Finally, a total about 135 chronic HD patients who signed their informed consent for this study were included. High-flux, polysulfone, disposable artificial kidneys were used (FX class dialyzer; Fresenius Medical Care, Bad Homburg, Germany). Basic demographic data and medical history were collected from medical records. Figure 1 depicts the flow chart of this study.

This cross-sectional study was approved by Research Ethics Committee, Hualien Tzu Chi Hospital, Buddhist Tzu Chi Medical Foundation (IRB108-182-B). Enrolled patients provided informed consent.

### 2.2. Anthropometric Analysis and Biochemical Investigations

Body mass index was calculated using height in meters and post-HD body weight in kilograms. Before HD, approximately 5 mL of blood was collected. Serum was obtained after 10 min centrifugation at 3000× *g*, and blood urea nitrogen, creatinine, albumin, total cholesterol, triglyceride, glucose, calcium, phosphorus, alkaline phosphatase, and C-reactive protein (CRP) levels were measured by an autoanalyzer (SiemensAdvia 1800, Siemens Healthcare GmbH, Henkestr, Germany) [14,15]. DM was defined as fasting plasma glucose >126 mg/dL or the use of oral hypoglycemic medications or insulin. Serum suPAR (Cloud-Clone Corp, Katy, TX, USA) and intact parathyroid hormone (iPTH, IBL International GmbH, Hamburg, Germany) were analyzed using commercially available enzyme immunoassay and enzyme-linked immunosorbent assay kit [14,15]. Right after HD, blood samples were also collected to calculate the fractional clearance index for urea (Kt/V) and urea reduction ratio in the single-compartment dialysis urea kinetic model.

### 2.3. Blood Pressure and Arterial Stiffness Measurements

We measured their brachial blood pressure three times at five-minute intervals after they had finished HD for thirty minutes. We averaged brachial systolic blood pressure (SBP) and brachial diastolic blood pressure (DBP). Hypertension was defined as brachial SBP ≥ 140 mmHg, brachial DBP ≥ 90 mmHg or having received any antihypertensive medication in the past two weeks. cfPWV needed electrocardiography (ECG) leads to record R wave interval and pressure applanation tonometry (SphygmoCor system, AtCor Medical, New South Wales, Australia) to record carotid and femoral artery pulse waves, as previously described [14,15]. Data were averaged from 10 consecutive cardiac cycles, and quality indices were calculated using integral software to minimize record errors. cfPWV is defined as the ratio of the length between carotid–femoral recording sites and the time lag of carotid–femoral pulse waves. The length was calculated by subtracting the distance between the carotid site and the sternal notch from the distance between the femoral site and the sternal notch. The augmentation index (AIx) is the augmentation pressure (systolic pressure minus the inflection pressure) divided by the pulse pressure (systolic minus diastolic pressure), expressed as a percentage. The calculated AIx75, AIx, was adjusted to a heart rate of 75/min, aortic SBP, and aortic DBP by SphygmoCor software 1.30 (Atcor Medical, Naperville, IL, USA). All participants lay supine in a quiet, thermostatic room for at least 10 min before cfPWV measurements [14,15]. According to the European Society of Hypertension and European Society of Cardiology guidelines [16], patients with a cfPWV of >10 m/s were assigned to the arterial stiffness group, and the rest made up the control group.

### 2.4. Statistical Analysis

We performed the Kolmogorov–Smirnov test to examine data distribution. HD duration, albumin, triglyceride, glucose, iPTH, alkaline phosphatase, CRP, and suPAR were logarithmically transformed for normality. Normally distributed data are expressed as mean ± standard deviation, and non-normally distributed data are expressed as median and interquartile ranges. To detect between-group differences, we applied the two-tailed Student’s independent t-test to normally distributed data and the Mann–Whitney U test to non-normally distributed data. We express data as percentages and differentiate using the χ2 test for categorical variables. In these variables, significantly associated with arterial stiffness, simple linear regression analysis, multiple logistic regression analysis, and multivariable forward stepwise regression analysis were performed. For log-transformed suPAR and variables, we examined their association using Spearman’s rank-order correlation coefficient. After confirming the effect of suPAR on arterial stiffness, we depicted the receiver operating curve to calculate the power using the area under the curve (AUC). Data were analyzed using IBM SPSS for Windows version 19.0 (IBM Corp., Armonk, NY, USA). A *p*-value less than 0.05 was statistically significant.

## 3. Results

Table 1 shows the clinical characteristics of the 135 chronic HD patients. Fifty-six patients had diabetes, and 74 patients had hypertension. Fifty-five patients were in the arterial stiffness group according to the cfPWV results. Significantly more patients in the arterial stiffness group had DM (*p* = 0.001) and hypertension (*p* = 0.039), as well as higher AIx75 (*p* = 0.003), higher aortic SBP (*p* = 0.034), and higher brachial SBP (*p* = 0.025), and they were older (*p* = 0.007). They also had more elevated glucose (*p* = 0.019), higher CRP (*p* = 0.039), and higher suPAR (*p* < 0.001) than the control group. There were no significant differences in sex and percentage of taking angiotensin receptor blockers, beta-blockers, calcium channel blockers, fibrate, or statins.

After adjusting for factors that showed association with arterial stiffness (DM, hypertension, age, aortic SBP, brachial SBP, glucose, CRP, and suPAR from Table 1), suPAR (odds ratio [OR]: 2.05; 95% confidence interval (CI): 1.58–2.70, *p* < 0.001), age (OR = 1.04; 95% CI 1.00–1.08, *p* = 0.046), and glucose (OR = 1.01; 95% CI 1.00–1.02, *p* = 0.007) predicted arterial stiffness in chronic HD patients after multivariable logistic regression analysis (Table 2).

**Figure 2:**
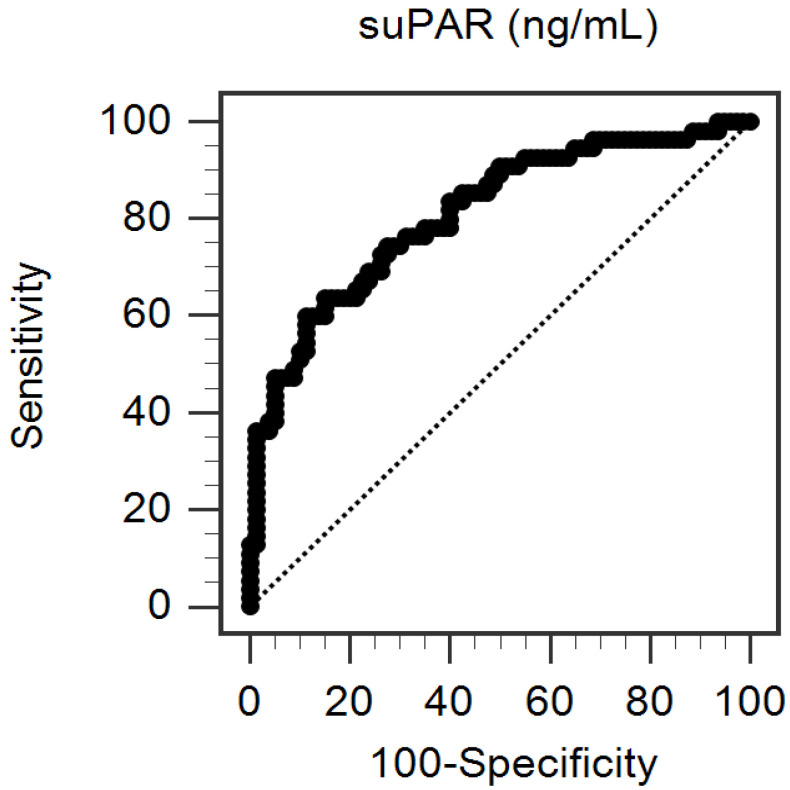
ROC curve for arterial stiffness prediction, determined by soluble urokinase-type plasminogen activator receptor (suPAR) level.

The correlations between cfPWV and clinical variables in HD patients are shown in Table 3. In the simple linear regression analysis, DM (*r* = 0.26; *p* = 0.003), age (*r* = 0.26, *p* = 0.002), aortic SBP (*r* = 0.23, *p* = 0.009), brachial SBP (*r* = 0.18, *p* = 0.041), log-glucose (*r* = 0.19, *p* = 0.023), log-CRP (*r* = 0.19, *p* = 0.024), and serum log-suPAR levels (*r* = 0.51; *p* < 0.001) are positively correlated with cfPWV. After we adjusted the above factors in the multivariable forward stepwise linear regression analysis, age (β = 0.17; adjusted R^2^ change = 0.023, *p* = 0.021), aortic SBP (β = 0.25; adjusted R^2^ change = 0.052, *p* = 0.001), and log-suPAR levels (β = 0.48, adjusted R^2^ change = 0.250, *p* < 0.001) had an independent and positive correlation with cfPWV in HD patients. The ROC curve for predicting arterial stiffness using suPAR demonstrated the AUC was 0.81 (95% CI 0.74–0.87, *p* < 0.001) (Figure 2). According to the Youden index, the optimal cut-off point of the suPAR level for predicting arterial stiffness was 4.85 pg/mL (sensitivity: 60.0%; specificity: 88.7%; positive predictive values: 77.5%; and negative predictive values: 76.3%).

The correlation between log-suPAR and variables was also explored by Spearman’s rank-order correlation coefficient (Table 4). DM (*p* = 0.047), age (*p* = 0.010), log-CRP (*p* < 0.001), AIx75 (*p* = 0.003), and cfPWV (*p* < 0.001) were positively associated with log-suPAR.

## 4. Discussion

This study shows that patients on HD and with high suPAR levels had a high risk of developing arterial stiffness. In addition, suPAR levels were found to be positively associated with DM, older age, CRP levels, AIx75, and cfPWV values in patients undergoing HD long term.

In recent decades, vascular injury and arterial stiffness measurements have become popular to predict CV diseases [8]. In cases of arterial stiffness, pulse-wave velocity measurement is a non-invasive and reproducible method. Some studies have found that the aorta correlated with CV risk more so than other muscular arteries, making cfPWV a gold standard of diagnosing aortic stiffness in patients undergoing HD [17]. Arterial stiffness refers to the loss of elasticity in the arterial walls, which can lead to increased wave reflection and a higher workload on the heart [18]. cfPWV is a non-invasive measure of arterial stiffness that has been shown to be a strong predictor of CV events and mortality [7,8]. Arterial stiffness is an important risk factor for the development of CV diseases, as it can lead to increased blood pressure, impaired blood flow, and damage to the arterial walls [17]. cfPWV is influenced by a variety of factors, including age, blood pressure, glucose levels, and inflammation [17,18]. Hypertension is a major risk factor for the development of arterial stiffness, as it can cause damage to the arterial walls and lead to increased stiffness [17,18]. DM is also associated with an increased risk of arterial stiffness, as elevated glucose levels can lead to the formation of advanced glycation end products that accumulate in the arterial walls and contribute to arterial stiffness [17,18]. In addition, the presence of inflammation, as indicated by elevated CRP levels, can further exacerbate arterial stiffness [17,18]. Besides using gold-standard cfPWV, we also measured the AIx75 as another arterial stiffness index. The AIx75 is derived from central artery tonometry and standardized heart rate to 75 beats per minute by software. It is associated with left ventricular hypertrophy [19], CV events [20], and mortality [21], independent of traditional risk factors. In this study, we also noted that chronic HD patients with arterial stiffness had higher aortic SBP, AIx75 levels, and cfPWV values, which are positively associated with aortic SBP after multivariable stepwise linear regression analyses. With increasing age, hyperglycemia, higher aortic blood pressure, brachial blood pressure, and inflammation often damage vessels, and many studies have shown that cfPWV values are positively associated with DM, hypertension, age, and CRP [18]. The present study showed similar findings. Furthermore, after adjusting for confounders, advanced age and higher blood glucose were found to be independent predictors of arterial stiffness development in patients on chronic HD.

Besides measuring cfPWV, numerous studies have investigated serum markers for predicting CV diseases and arterial stiffness. Urokinase plasminogen activator receptor (uPAR), a glycosylphosphatidylinositol-anchored protein on the cell surface, has been recognized as a potential marker of atherosclerosis [22]. In acute and chronic inflammation, uPAR is dislodged from the cell surface, promoting coagulation and immune activation. The accumulation of monocyte-derived macrophage-foam cells in atherosclerotic plaques express high levels of uPAR [23]. suPAR correlated with other inflammation markers such as CRP [24], procalcitonin [24], erythrocyte sedimentation rate [25], and many cytokines in acute and chronic diseases [26]. suPAR increases with age and is demonstrated to predict incident DM [27], CV diseases [28], and CKD progression [29]. Our data are compatible with these results, and suPAR levels are positively associated with DM, older age, and CRP levels in patients on chronic HD.

Arterial stiffness, an early pathology of atherosclerosis, and suPAR, a serum marker in CV diseases, have been studied in different populations. The SAfrEIC study measured suPAR and arterial stiffness in Africans and Caucasians [30]. Although a single regression analysis revealed a correlation between suPAR and arterial stiffness, such correlation was lost in the subgroup analysis. In patients with COPD [31] and type I DM [32], suPAR correlated with arterial stiffness in the univariable and multivariable analyses. Our study of patients undergoing HD, similar to patients with COPD and type 1 DM, showed a similar association between arterial stiffness and suPAR, and suPAR levels are positively associated with cfPWV levels and AIx75 levels in HD patients.

This study has several limitations. First, this is a single-center cross-sectional study; thus, it may be difficult to generalize the results to all patients on HD, even the general population. To strengthen the relationship, analyzing larger samples and an extended follow-up period may be helpful. Second, although suPAR is less likely affected by acute illness, a mild infection may disrupt actual relationships. Evaluating more acute phase proteins such as IL-6, IL-10, and IL-18 may reduce the discrepancy. Third, emerging results show that home blood pressure has a stronger association with cfPWV than post-dialysis blood pressure. However, we did not collect data on home blood pressure. Finally, we took all these arterial stiffness indexes and blood pressure after HD. The relation between cfPWV, AIx75, aortic blood pressure and suPAR is unclear. A study by Erdan et al. indicates that these indexes decreased after dialysis [33]. However, another study from Georgianos et al. shows stable cfPWV after dialysis [34]. Because the arterial stiffness cutoff value is based on the general normovolemic population, measuring arterial stiffness before HD may underestimate the true effect of suPAR on arterial stiffness. In this study, we did not measure arterial stiffness parameters before dialysis. Further additional longitudinal studies with large samples and collecting evidence on these diseases will help us clarify their relationship.

## 5. Conclusions

Along with old age and glucose, our study showed a positive relationship between arterial stiffness and suPAR in patients on chronic HD; however, the mechanism must be further examined.

## Figures and Tables

**Table 1:** Clinical variables of the 135 hemodialysis patients in the control and arterial stiffness group.

Variables	All Patients (*n* = 135)	Control Group (*n* = 80)	Arterial Stiffness Group (*n* = 55)	*p* Value
Age (years)	62.8 ± 13.3	60.2 ± 13.6	66.4 ± 12.1	0.007 *
HD vintage (months)	56.2 (23.5–123.6)	65.9 (21.8–132.9)	53.4 (25.8–104.6)	0.483
Height (cm)	159.9 ± 8.4	159.5 ± 8.6	160.5 ± 8.1	0.524
Pre-HD BW (Kg)	63.6 ± 14.8	62.9 ± 15.7	64.5 ± 13.5	0.551
Post-HD BW (Kg)	61.4 ± 14.3	60.8 ± 15.2	62.2 ± 13.1	0.559
BMI (Kg/m^2^)	25.00 ± 5.0	24.8 ± 5.4	25.3 ± 4.5	0.556
Aortic SBP (mmHg)	133.8 ± 26.0	129.9 ± 25.8	139.6 ± 25.5	0.034 *
Aortic DBP (mmHg)	79.5 ± 18.5	79.5 ± 18.8	79.5 ± 18.1	0.993
Carotid–femoral PWV (m/s)	9.4 ± 2.1	7.9 ± 1.1	11.5 ± 1.0	<0.001 *
AIx75 (%)	31.3 ± 6.7	30.0 ± 7.2	33.4 ± 25.2	0.003 *
Brachial SBP (mmHg)	144.0 ± 27.0	139.7 ± 27.3	150.2 ± 25.4	0.025 *
Brachial DBP (mmHg)	78.1 ± 16.7	78.3 ± 16.2	77.8 ± 17.7	0.861
Albumin (g/dL)	4.1 (3.9–4.4)	4.1 (3.9–4.4)	4.1 (3.9–4.3)	0.397
Total cholesterol (mg/dL)	145.3 ± 34.5	148.1 ± 38.0	141.2 ± 28.6	0.257
Triglyceride (mg/dL)	117.0 (84.0–178.0)	110.5 (83.3–199.5)	122.0 (89.0–175.0)	0.722
Glucose (mg/dL)	132.0 (105.0–173.0)	127.5 (103.0–151.0)	145.0 (111.0–194.0)	0.019 *
BUN (mg/dL)	61.4 ± 15.0	60.7 ± 14.2	62.5 ± 16.0	0.497
Creatinine (mg/dL)	9.37 ± 2.05	9.43 ± 2.09	9.28 ± 2.00	0.675
Total calcium (mg/dL)	9.01 ± 0.78	8.92 ± 0.73	9.14 ± 0.83	0.108
Phosphorus (mg/dL)	4.72 ± 1.29	4.75 ± 1.32	4.66 ± 1.26	0.696
iPTH (pg/mL)	222.9 (126.7–410.1)	249.4 (130.3–449.4)	192.9 (118.5–374.2)	0.298
Alkaline phosphatase (IU/L)	76.0 (61.0–107.0)	77.0 (62.0–102.0)	76.0 (60.0–113.0)	0.757
suPAR (ng/mL)	3.13 (2.29–5.30)	2.54 (2.07–3.58)	5.14 (3.16–7.87)	<0.001 *
C-reactive protein (mg/dL)	0.30 (0.08–0.95)	0.23 (0.05–0.92)	0.45 (0.18–1.01)	0.039 *
Urea reduction rate	0.73 ± 0.04	0.73 ± 0.05	0.73 ± 0.04	0.936
Kt/V (Gotch)	1.34 ± 0.17	1.34 ± 0.18	1.34 ± 0.16	0.966
Female, *n* (%)	66 (48.9)	42 (52.5)	24 (43.6)	0.311
Diabetes mellitus, *n* (%)	56 (41.5)	24 (30.0)	32 (58.2)	0.001 *
Hypertension, *n* (%)	74 (54.8)	38 (47.5)	36 (65.5)	0.039 *
Angiotensin receptor blocker, *n* (%)	39 (28.9)	20 (25.0)	19 (34.5)	0.229
β-blocker, *n* (%)	42 (31.1)	22 (27.5)	20 (36.4)	0.274
Calcium channel blocker, *n* (%)	51 (37.8)	27 (33.8)	24 (43.6)	0.244
Arteriovenous fistula, *n* (%)	96 (71.1)	56 (70.0)	40 (72.7)	0.552
Arteriovenous graft, *n* (%)	36 (26.7)	23 (63.9)	13 (36.1)	
Perm catheter, *n* (%)	3 (2.2)	1 (1.3)	2 (3.6)	

Normally distributed continuous variables are shown as mean ± standard deviation; variables not normally distributed are shown as median and interquartile range; categorical variables are presented as number (%). *p*-values are obtained by the Student’s t-test in normally distributed variables, by the Mann–Whitney U test in non-normally distributed variables, and by the chi-square test in categorical variables. HD, hemodialysis; BW, body weight; BMI, body mass index; PWV, pulse wave velocity; AIx75, augmentation index at a heart rate of 75 bpm; SBP, systolic blood pressure; DBP, diastolic blood pressure; BUN, blood urea nitrogen; iPTH, intact parathyroid hormone; suPAR, soluble urokinase-type plasminogen activator receptor; Kt/V, fractional clearance index for urea. * *p* value refers to the comparison between the arterial stiffness group and the control group.

**Table 2:** Factors correlated to arterial stiffness, determined by multivariable logistic regression analysis.

Variables	Odds Ratio	95% Confidence Interval	*p* Value
suPAR, 1 ng/mL	2.05	1.58–2.70	<0.001 *
Age, 1 year	1.04	1.00–1.08	0.046 *
Glucose, 1 mg/dL	1.01	1.00–1.02	0.007 *
C-reactive protein, 0.1 mg/dL	0.98	0.94–1.02	0.243
Aortic SBP, 1 mmHg	1.04	1.00–1.09	0.055
Brachial SBP, 1 mmHg	0.97	0.94–1.02	0.209
Diabetes mellitus (present)	2.50	0.76–5.53	0.155
Hypertension (present)	1.94	0.65–5.82	0.235

The multivariable logistic regression analysis was performed using various factors (diabetes mellitus, hypertension, age, aortic SBP, brachial SBP, glucose, C-reactive protein, and suPAR). SBP, systolic blood pressure; suPAR, soluble urokinase-type plasminogen activator receptor. * *p* < 0.05 was statistically significant.

**Table 3:** Factors correlated to carotid–femoral pulse wave velocity, determined by linear regression analysis.

Variables	Carotid–Femoral Pulse Wave Velocity (m/s)				
	Simple Linear Regression		Multivariable Linear Regression		
	*r*	*p* Value	Beta	Adjusted R^2^ Change	*p* Value
Female	−0.12	0.162	—	—	—
Diabetes mellitus	0.26	0.003 *	—	—	—
Hypertension	0.16	0.057	—	—	—
Age (years)	0.26	0.002 *	0.17	0.023	0.021 *
Log-HD vintage (months)	−0.14	0.096	—	—	—
Height (cm)	0.05	0.587	—	—	—
Pre-HD BW (Kg)	0.10	0.265	—	—	—
BMI (Kg/m^2^)	0.11	0.202	—	—	—
Aortic SBP (mmHg)	0.23	0.009 *	0.25	0.052	0.001 *
Aortic DBP (mmHg)	0.06	0.493	—	—	—
Brachial SBP (mmHg)	0.18	0.041 *	—	—	—
Brachial DBP (mmHg)	0.05	0.592	—	—	—
Log-Albumin (g/dL)	−0.07	0.453	—	—	—
TCH (mg/dL)	−0.08	0.339	—	—	—
Log-TG (mg/dL)	0.03	0.704	—	—	—
Log-Glucose (mg/dL)	0.20	0.023 *	—	—	—
BUN (mg/dL)	0.02	0.800	—	—	—
Creatinine (mg/dL)	−0.002	0.978	—	—	—
Total calcium (mg/dL)	0.07	0.448	—	—	—
Phosphorus (mg/dL)	0.02	0.794	—	—	—
Log-iPTH (pg/mL)	−0.09	0.297	—	—	—
Log-ALP (IU/L)	−0.03	0.758	—	—	—
Log-suPAR (pg/mL)	0.51	<0.001 *	0.48	0.250	<0.001 *
Log-CRP (mg/dL)	0.19	0.024 *	—	—	—
Urea reduction rate	−0.03	0.738	—	—	—
Kt/V (Gotch)	−0.03	0.716	—	—	—

HD vintage, albumin, TG, glucose, iPTH, ALP, CRP, and suPAR were in skewed distribution and were log-transformed before analysis. We perform simple linear regression analyses and multivariable stepwise linear regression analyses (adopted factors: diabetes mellitus, age, aortic SBP, brachial SBP, log-glucose, log-CRP, and log-suPAR). HD, hemodialysis; BW, body weight; BMI, body mass index; SBP, systolic blood pressure; DBP, diastolic blood pressure; TCH, total cholesterol; TG, triglyceride; BUN, blood urea nitrogen; iPTH, intact parathyroid hormone;ALP, alkaline phosphatase; suPAR; soluble urokinase-type plasminogen activator receptor; CRP, C-reactive protein; Kt/V, fractional clearance index for urea. * *p* < 0.05 was statistically significant.

**Table 4:** Correction between log-transformed soluble urokinase-type plasminogen activator receptor level and clinical variables.

Variables	Spearman’s Correlation Coefficient	*p* Value
Female	0.020	0.815
Diabetes mellitus	0.171	0.047 *
Hypertension	0.032	0.709
Age (years)	0.222	0.010 *
Log-HD vintage (months)	−0.001	0.994
Height (cm)	−0029	0.740
Pre-HD BW (Kg)	0.029	0.741
BMI (Kg/m^2^)	0.064	0.463
Aortic SBP (mmHg)	−0.025	0.774
Aortic DBP (mmHg)	−0.086	0.322
Brachial SBP (mmHg)	0.042	0.632
Brachial DBP (mmHg)	−0.127	0.142
Log-Albumin (g/dL)	0.074	0.391
TCH (mg/dL)	0.074	0.391
Log-TG (mg/dL)	0.132	0.128
Log-Glucose (mg/dL)	0.047	0.589
BUN (mg/dL)	0.124	0.153
Creatinine (mg/dL)	−0.015	0.860
Total calcium (mg/dL)	0.134	0.120
Phosphorus (mg/dL)	0.022	0.804
Log-iPTH (pg/mL)	−0.119	0.169
Log-ALP (IU/L)	−0.018	0.837
Log-CRP (mg/dL)	0.324	<0.001 *
Urea reduction rate	0.053	0.538
Kt/V (Gotch)	0.036	0.680
Carotid–femoral PWV (m/s)	0.506	<0.001 *
AIx75 (%)	0.250	0.003 *

HD duration, albumin, triglyceride, glucose, iPTH, ALP, CRP, and suPAR were in skewed distribution and were log-transformed before analysis. HD, hemodialysis; BW, body weight; BMI, body mass index; PWV, pulse wave velocity; SBP, systolic blood pressure; DBP, diastolic blood pressure; TCH, total cholesterol; TG, triglyceride; BUN, blood urea nitrogen; iPTH, intact parathyroid hormone; ALP, alkaline phosphatase; suPAR, soluble urokinase-type plasminogen activator receptor; CRP, C-reactive protein; Kt/V, fractional clearance index for urea; AIx75, augmentation index at a heart rate of 75 bpm. * *p* < 0.05 was considered significant.

## Data Availability

The data presented in this study are available on request from the corresponding author.
